# Neurodevelopmental Impairment in Children After Group B Streptococcal Disease Worldwide: Systematic Review and Meta-analyses

**DOI:** 10.1093/cid/cix663

**Published:** 2017-11-06

**Authors:** Maya Kohli-Lynch, Neal J Russell, Anna C Seale, Ziyaad Dangor, Cally J Tann, Carol J Baker, Linda Bartlett, Clare Cutland, Michael G Gravett, Paul T Heath, Margaret Ip, Kirsty Le Doare, Shabir A Madhi, Craig E Rubens, Samir K Saha, Stephanie Schrag, Ajoke Sobanjo-ter Meulen, Johan Vekemans, Catherine O’Sullivan, Firdose Nakwa, Hechmi Ben Hamouda, Habib Soua, Kyriaki Giorgakoudi, Shamez Ladhani, Theresa Lamagni, Hilary Rattue, Caroline Trotter, Joy E Lawn

**Affiliations:** 1 Maternal, Adolescent, Reproductive and Child Health Centre, London School of Hygiene & Tropical Medicine, United Kingdom;; 2 Centre for Child and Adolescent Health, School of Social and Community Medicine, University of Bristol, United Kingdom;; 3 King’s College London, United Kingdom;; 4 College of Health and Medical Sciences, Haramaya University, Dire Dawa, Ethiopia;; 5 Medical Research Council, Respiratory and Meningeal Pathogens Research Unit,; 6 Department of Science and Technology/National Research Foundation, Vaccine Preventable Diseases, and; 7 Department of Paediatrics, Faculty of Health Sciences, University of the Witwatersrand, Johannesburg, South Africa;; 8 Neonatal Medicine, University College London Hospitals NHS Foundation Trust, United Kingdom;; 9 Departments of Pediatrics and Molecular Virology and Microbiology, Baylor College of Medicine, Houston, Texas;; 10 Department of International Health, Johns Hopkins Bloomberg School of Public Health, Baltimore, Maryland;; 11 Global Alliance to Prevent Prematurity and Stillbirth, Seattle, Washington;; 12 Department of Obstetrics and Gynecology, University of Washington, Seattle;; 13 Vaccine Institute, Institute for Infection and Immunity, St George’s Hospital, University of London and St George’s University Hospitals NHS Foundation Trust, United Kingdom;; 14 Department of Microbiology, Faculty of Medicine, Chinese University of Hong Kong;; 15 Centre for International Child Health, Imperial College London, United Kingdom;; 16 National Institute for Communicable Diseases, National Health Laboratory Service, Johannesburg, South Africa;; 17 Department of Global Health, University of Washington, Seattle;; 18 Bangladesh Institute of Child Health, Dhaka;; 19 National Center for Immunization and Respiratory Diseases, Centers for Disease Control and Prevention, Atlanta, Georgia;; 20 Bill & Melinda Gates Foundation, Seattle, Washington;; 21 World Health Organization, Geneva, Switzerland;; 22 Department of Neonatology, University Hospital Tahar Sfar, Mahdia, Tunisia;; 23 City University, United Kingdom;; 24 Public Health England, London, United Kingdom; and; 25 University of Cambridge, United Kingdom

**Keywords:** Group B *Streptococcus*, impairment, infants, disability, estimate

## Abstract

**Background:**

Survivors of infant group B streptococcal (GBS) disease are at risk of neurodevelopmental impairment (NDI), a burden not previously systematically quantified. This is the 10th of 11 articles estimating the burden of GBS disease. Here we aimed to estimate NDI in survivors of infant GBS disease.

**Methods:**

We conducted systematic literature reviews (PubMed/Medline, Embase, Latin American and Caribbean Health Sciences Literature [LILACS], World Health Organization Library Information System [WHOLIS], and Scopus) and sought unpublished data on the risk of NDI after invasive GBS disease in infants <90 days of age. We did meta-analyses to derive pooled estimates of the percentage of infants with NDI following GBS meningitis.

**Results:**

We identified 6127 studies, of which 18 met eligibility criteria, all from middle- or high-income contexts. All 18 studies followed up survivors of GBS meningitis; only 5 of these studies also followed up survivors of GBS sepsis and were too few to pool in a meta-analysis. Of meningitis survivors, 32% (95% CI, 25%–38%) had NDI at 18 months of follow-up, including 18% (95% CI, 13%–22%) with moderate to severe NDI.

**Conclusions:**

GBS meningitis is an important risk factor for moderate to severe NDI, affecting around 1 in 5 survivors. However, data are limited, and we were unable to estimate NDI after GBS sepsis. Comparability of studies is difficult due to methodological differences including variability in timing of clinical reviews and assessment tools. Follow-up of clinical cases and standardization of methods are essential to fully quantify the total burden of NDI associated with GBS disease, and inform program priorities.

## Teaser

Group B *Streptococcus* (GBS) is a leading cause of invasive disease in infants, causing mortality and neurodevelopmental impairment (NDI) in survivors. This article estimates the percentage of survivors of infant GBS disease with NDI.

During the Millennium Development Goal era, there have been considerable achievements in child survival worldwide, although progress for neonatal deaths (days 0–27) has been slower [1, 2]. In the era of the Sustainable Development Goals, there is continued emphasis on survival, to end preventable newborn and child deaths, but also to move beyond survival to improved health and maximized child well-being [3]. This shift is highlighted in the World Health Organization (WHO) Global Strategy for Women’s, Children’s, and Adolescents’ Health, and the 3 pillars of survive, thrive, and transform [4]. As maternal and newborn care improves in low- and middle-income countries, there may be an increase in neurodevelopmental impairment (NDI), including in survivors of invasive infection, as was observed in high-income countries following advances in perinatal care [5, 6].

Infection is an important cause of neonatal and infant death [1]. What is less well recognized is the burden of NDI in survivors of infant infection. NDI may follow invasive infections including pneumonia, sepsis, and meningitis [7]. The neuroimaging findings, mechanisms of brain injury, and subsequent NDI after invasive infectious disease are outlined in Box 1. Data are insufficient to estimate the risk after invasive neonatal disease other than meningitis, after which around 23% (95% confidence interval [CI], 19%–26%) of survivors are estimated to have moderate to severe NDI [7]. Intrauterine and neonatal insults, including infections, are estimated to cause NDI in 39% (interquartile range [IQR], 20%–55%) of survivors [8].

**Box 1. Neuroimaging, Mechanisms of Brain Injury, and Neurodevelop mental Impairment After Infant Meningitis and Sepsis**

Meningitis and sepsis can cause brain injury in term and preterm infants. Magnetic resonance imaging (MRI) findings consistently show cerebrovascular involvement, and abnormal findings on neonatal MRI have been clearly associated with poor neurodevelopmental outcome at 2 years [9]. A retrospective cohort study in France found that all 9 cases of infant GBS meningitis in term babies had abnormal findings on MRI, of which 56% showed ischemic infarction (neonatal stroke). Furthermore, a case series of 8 term infants with GBS meningitis and ischemic stroke on MRI found 2 recognizable patterns of injury, deep perforator arterial infarction, and more superficial cortical injury [10, 11]. Case reports of neuroimaging in term infants with GBS meningitis have also found severe global cerebral vasculopathy and transverse myelitis [12, 13]. In preterm infants, inflammatory cytokines associated with infection increase the permeability of the blood–brain barrier and have an adverse effect on myelin and myelin-producing cells, resulting in periventricular leukomalacia, a condition strongly associated with neurodisability [14, 15]. In addition, sepsis causes brain injury indirectly through disseminated intravascular coagulopathy and hypotension; this is important to consider in cases of neonatal sepsis without meningitis [15]. Furthermore, bloodstream infection can have a sensitizing effect in the development of hypoxic-ischemic encephalopathy, which is also associated with neurodevelopmental impairment [16].

Group B *Streptococcus* (GBS; *Streptococcus agalactiae*) is a leading cause of infant sepsis and meningitis. Its contribution to NDI and associated disability has not, however, previously been assessed by a comprehensive systematic review or pooled estimates. The long-term consequences of infant GBS disease are important to understand, to estimate the full burden of GBS disease, including in terms of standard measures used to assess public health priorities, such as disability-adjusted life-years (DALYs).

**Figure 1.**
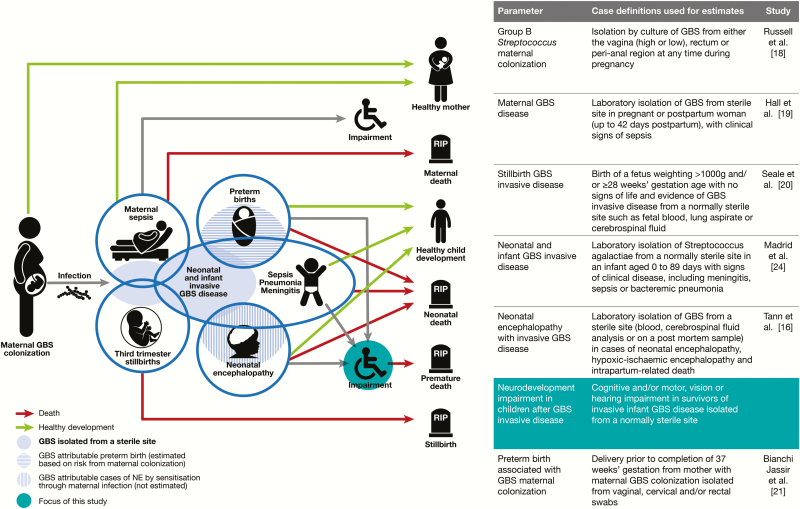
Neurodevelopmental impairment after infant group B streptococcal (GBS) disease in the disease schema for GBS, as described by Lawn et al [17]. Abbreviations: GBS, group B *Streptococcus*; NE, neonatal encephalopathy.

We aimed to estimate the percentage of survivors of infant GBS disease with NDI (Figure 1) as part of a supplement estimating the burden of GBS disease in pregnant women, stillbirths, and infants, which is important in terms of public health policy, particularly vaccine development, as outlined elsewhere in this supplement [17]. The supplement includes systematic reviews and meta-analyses on GBS colonization and adverse outcomes associated with GBS around birth [16,18–24]. These are reported individually according to international guidelines [25, 26] and are used for estimates of the burden of GBS worldwide [27] (Figure 1).

## OBJECTIVES

1. To provide a comprehensive, systematic literature review and meta-analyses to assess the following parameters: (*i*) percentage of survivors with any NDI after infant GBS disease; (*ii*) percentage of survivors with moderate to severe NDI after infant GBS disease.
2. To assess these data for input to estimate the burden of GBS in pregnancy, stillbirth, and infants.
3. To evaluate the data gaps and make recommendations to improve the data on NDI after infant GBS disease.

## METHODS

This article is part of a study entitled “Systematic estimates of the global burden of GBS worldwide in pregnant women, stillbirths and infants.” It was submitted for ethical approval to the London School of Hygiene & Tropical Medicine (reference number 11966) and approved on 30 November 2016.

### Exposure

Case definitions for invasive infant GBS disease include GBS meningitis (clinical signs of possible serious bacterial infection and cerebrospinal fluid (CSF) culture /polymerase chain reaction (PCR)/latex agglutination positive for GBS or blood culture/PCR/latex agglutination positive for GBS with CSF leukocyte count of >20 × 10^6^/L), GBS sepsis (clinical signs of possible serious bacterial infection and blood culture/PCR/latex agglutination positive for GBS), and GBS pneumonia. These definitions are fully described in Supplementary Table 1a.

### Outcome

The outcome of interest is NDI. Impairment is defined as a problem in body function and structure such as significant deviation or loss (Supplementary Table 2) [28]. For this analysis of NDI, we combined the specific definitions used by the Global Burden of Disease study 2013 (GBD2013) [29] and others [30] to be consistent with the literature. Therefore, NDIs are categorized as intellectual and/or motor, vision, or hearing impairment and severity classified as mild, moderate, or severe. To maintain consistency with GBD2013 definitions, we did not include social, language, and behavioral NDI in the quantitative analysis for this review. Disability weights for each impairment domain and severity used in GBD2013 are also listed in Supplementary Table 1b.

### Data Searches and Inputs

We identified data through systematic review of the published literature and through development of an investigator group surveying clinicians, researchers, and relevant professional institutions worldwide. For this paper, we did systematic literature searches of PubMed/Medline, Embase, the World Health Organization Library Information System (WHOLIS), Literature in the Health Sciences in Latin America and the Caribbean (LILACS), and Scopus until January 2017. We searched databases with variants of terms related to “child,” “disability,” “impairment,” “GBS,” “morbidity,” and “mortality.” Medical subject heading (MeSH) terms were used where possible (see Supplementary Table 3 for the full list of search terms). There were no date and/or language restrictions applied and we translated texts to English when published in other languages. We used snowball searches of article reference lists to identify additional studies. We requested unpublished data from an international network of investigators. Where reporting of child neurodevelopmental outcomes was not clear, or outcomes were not reported separately for GBS, we contacted authors for clarification of terms and definitions used, and assessment of survivors of infant GBS disease. If authors were uncontactable, 2 clinicians (M. K. L., N. R.) discussed and came to a consensus to classify impairment. We matched NDI outcomes to GBD2013 and *International Classification of Diseases* [31] definitions to ensure optimal comparability between studies.

**Figure 2.**
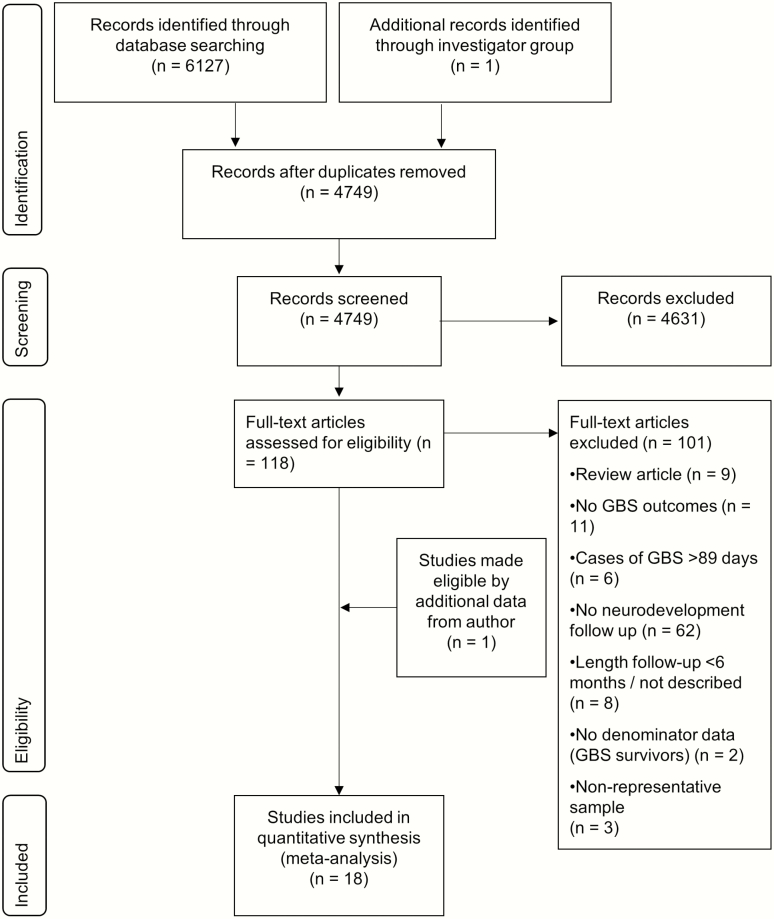
Data search and selection. Abbreviation: GBS, group B *Streptococcus*.

The full search strategy is illustrated in Figure 2 [26]. Two independent investigators (M. K. L., N. R.) performed the database searches, screened titles for duplicates and for eligibility, and screened abstracts to assess their suitability for inclusion, and both reviewers extracted data. Where there was discrepancy between the 2 reviewers, a third investigator (A. S.) made the final decision.

### Inclusion and Exclusion Criteria

We included studies of infants with GBS disease in the first 90 days after birth (see case definitions), which reported study characteristics and assessed child neurodevelopmental outcomes at a median of ≥6 months of age. Studies with nonrepresentative samples of cases (eg, with a selection bias of preterm births) and unsuitable article types were excluded (Supplementary Table 4). Only cases of moderate or severe NDI were included in the main meta-analysis as there was lack of consistency in definition and proportions of survivors with mild or profound NDI.

The quality of the studies and risks of bias were assessed using inclusion and exclusion criteria (see Supplementary Table 4) on validity of study methods, interpretation of results, and applicability of the results.

### Data Collection

Data on study characteristics and results were extracted into prespecified Excel abstraction forms, and then imported to Stata 14 software (StataCorp, La Jolla, California) for meta-analyses.

Meta-analyses were done to assess risk of NDI by severity, length of follow-up, and neonatal mortality rate (NMR) (per 1000 live births) context.

We used random-effects meta-analyses to estimate risk of NDI using the DerSimonian and Laird method [32]. The primary meta-analysis was of GBS meningitis survivors followed up for a median of ≥18 months with moderate to severe NDI. We decided to focus on moderate to severe impairment as more consistent results are seen with moderate to severe NDI [33] and there was lack of consistency in the definition and proportions of survivors with mild NDI. We chose a median follow-up of ≥18 months to include as many studies as possible while demonstrating the most accurate possible estimate of NDI, and cases of NDI are likely to manifest with longer-term follow-up. A threshold NMR of 5 per 1000 live births was used for the sensitivity analyses to reflect newborn care context as high-income countries have an average NMR of 4 per 1000 [34]. Given lack of variation with varying NMR settings (see below), we did not report the primary meta-analysis by NMR setting.

The following sensitivity meta-analyses were done to assess the effect of length of follow-up, severity of NDI, and NMR setting: (1) GBS meningitis survivors followed up for a median of ≥18 months with any NDI; (2) GBS meningitis survivors followed up for a median of ≥18 months with moderate to severe NDI where NMR was ≥5 per 1000 live births; (3) GBS meningitis survivors followed up for a median of ≥18 months with moderate to severe NDI where NMR was <5 per 1000; (4) GBS meningitis survivors followed up for a median of ≥6 months with any NDI; (5) GBS meningitis survivors followed up for a median of ≥6 months with moderate to severe NDI; (6) GBS meningitis survivors followed up for a median of ≥6 months with moderate to severe NDI where NMR was ≥5 per 1000; (7) GBS meningitis survivors followed up for a median of ≥6 months with moderate to severe NDI where NMR was <5 per 1000.

## RESULTS

### Literature Search

We identified 6127 studies through database searches. Of these, 118 full texts were reviewed and 16 studies met the inclusion criteria (Figure 2) [35–49]. Two unpublished datasets (Heath et al and Dangor et al) were provided by the investigator group, 1 of which contained longer-term NDI outcomes of a cohort described in a published article. Overall, 18 studies were included in the quantitative analysis.

### Study Characteristics

All 18 studies followed up survivors of infant GBS meningitis; 5 of these studies also followed up survivors of infant GBS sepsis (Table 1). The numbers of GBS sepsis cases and survivors assessed were too few to pool in a meta-analysis (see Supplementary Table 5 for summary of results); therefore, we have meta-analyzed NDI outcomes after infant GBS meningitis only.

**Table 1.** Characteristics of Included Studies Investigating Neurodevelopmental Outcomes After Infant Group B Streptococcal Meningitis

UN Region	UN Subregion	Country	Author	Publication Year	Followed Sepsis Cases (Yes/No)	Median Year of Data Collection	NMR (per 1000 Live Births)	Facility	Minimum Median Follow-up, y	No. of ND Assess- ments	No. of GBS Meningitis Survivors (% of GBS Meningitis Cases)
Africa	Southern Africa	South Africa	Dangor (unpublished)	…^a^	Y	2014	11	Hospital	1	3	30 (85.7%)
	Northern Africa	Tunisia	Ben Hamouda [36]	2013	N	2003	15	Hospital	5	1	7 (70.0%)
Americas	Northern America	US	Libster [45]	2012	N	2002	5	Hospital	6.8	1	85 (94.4%)
		US	Franco [42]	1992	N	1975	12	Hospital	4.5	19	10 (90.9%)
		US	Wald [47]	1986	N	1973	13	Hospital	10.5	1	54 (73.0%)
		US	Chin [40]	1985	N	1978	9	Hospital	4.3	5	21 (77.8%)
		US	Edwards [41]	1985	N	1976	10	Hospital	6	1	48 (78.7%)
		US	Haslam [43]	1977	N	1971	14	Hospital	3.6	1	15 (83.3%)
		US	Horn [44]	1974	Y	Unknown	12	Hospital	1.6	1	7 (41.2%)
		US	Baker [35]	1973	N	1971	14	Hospital	0.5	1	23 (69.7%)
Asia	Southeastern Asia	Singapore	Wee [48]	2016	N	2005	1	Hospital	2	4	20 (95.2%)
	Eastern Asia	China	Zhu [49]	2014	N	2009	9	Hospital	1.9	1	11 (84.6%)
Europe	Northern Europe	UK	Heath (unpublished)	…	Y	2015	2	Hospital	3	1	37 (unknown as study ongoing)
		UK	Bedford [37]	2001	N	1986	5	National surveillance	5	1	103 (unknown)
		Sweden	Bennhagen [38]	1987	N	(a) 1979; (b) 1983	(a) 6; (b) 4	National surveillance	(a) 1.5 (b) 1.5	(a) 1; (b) 1	(a) 16 (80.0%); (b) 9 (100.0%)
		Denmark	Carstensen [39]	1985	Y	1980	6	National surveillance	0.75	Not reported	26 (74.3%)
	Western Europe	Germany	Schroder [46]	1982	Y	1975	9	Hospital	2.3	1	10 (unknown)
										Total	532

Abbreviations: GBS, group B *Streptococcus*; ND, neurodevelopmental; NMR, neonatal mortality rate per 1000 live births; UK, United Kingdom; UN, United Nations; US, United States.

^a^Three- and 6-month neurodevelopmental follow-up assessment results have been published [50].

**Figure 3.**
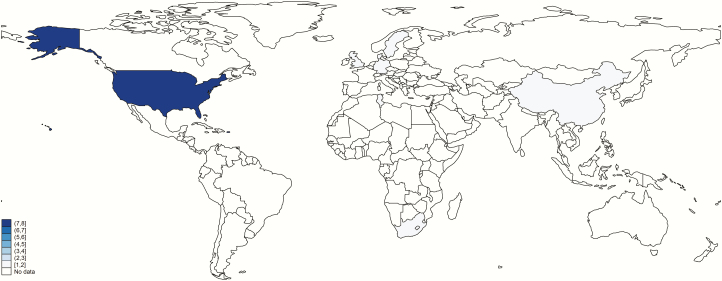
Geographic spread of inputs for neurodevelopmental impairment after infant Group B *Streptococcus* meningitis. Borders of countries/territories in map do not imply any political statement.

Ten of the 18 articles included were published between 1973 and 1992. The remaining 8 studies were from 2000 onward. One article [38] contained datasets from 2 periods (1976 and 1983) and were entered into the meta-analyses separately. Years of data collection ranged from 1970 to 2017, with the median years of data collection ranging from 1971 to 2016. Most (8/18) studies were done in the United States, 2 in the United Kingdom, 2 in Sweden, and 1 each in Germany, Denmark, Singapore, China, Tunisia, and South Africa, the latter 3 being the only studies conducted in middle-income contexts (Figure 3). The majority (15/18) of studies were carried out in hospitals, while 3 of 18 studies used national surveillance data. The median length of follow-up of infant GBS meningitis survivors ranged from 6 months to 10.5 years. Neonatal mortality rates at studies’ median point of data collection ranged from 1–15 per 1000 live births [51–54]. Further detailed characteristics of studies included after systematic review for NDI following infant GBS meningitis are given in Table 2.

**Table 2.** Results of Meta-analyses for Sensitivity Testing to Assess Variation With Length of Follow-up and/or Severity of Neurodevelopmental Outcome by Neonatal Mortality Rate Setting

Meta-analysis	Population Included	No. of Studies (No. of Children Followed up)	Percentage of GBS Meningitis Survivors With Neurodevelopmental Impairment (95% CI)
1.	GBS meningitis survivors followed up for median of ≥18 mo with any NDI	15 (453)	32 (25–38)
2.	GBS meningitis survivors followed up for median of ≥18 mo with moderate to severe NDI where NMR ≥5/1000	12 (387)	18 (12–24)
3.	GBS meningitis survivors followed up for median of ≥18 mo with moderate to severe NDI where NMR <5/1000	3 (66)	18 (8–28)
4.	GBS meningitis survivors followed up for median of ≥6 mo with any NDI	18 (532)	27 (20–34)
5.	GBS meningitis survivors followed up for median of ≥6 mo with moderate to severe NDI	18 (532)	15 (11–20)
6.	GBS meningitis survivors followed up for median of ≥6 mo with moderate to severe NDI where NMR ≥5/1000	15 (466)	15 (10–20)
7.	GBS meningitis survivors followed up for median of ≥6 mo with moderate to severe NDI where NMR <5/1000	3 (66)	18 (8–28)

Abbreviations: CI, confidence interval; GBS, group B Streptococcus; NDI, neurodevelopmental impairment; NMR, neonatal mortality rate per 1000 live births.

A wide range of neurodevelopment assessment methods were applied. In total, 44 different methods were used (Supplementary Table 6); 31 methods were used to assess child cognitive, motor, language, and socioemotional or behavioral development; 8 methods to assess vision; and 9 methods to assess hearing. Not all studies covered all domains or described the methods (Supplementary Table 6). The number of child development assessments done for each child ranged from 1 to 19 (Supplementary Table 6).

The primary meta-analysis was for risk of moderate to severe NDI in survivors of infant GBS meningitis at a median ≥18 months of follow-up.

**Figure 4.**
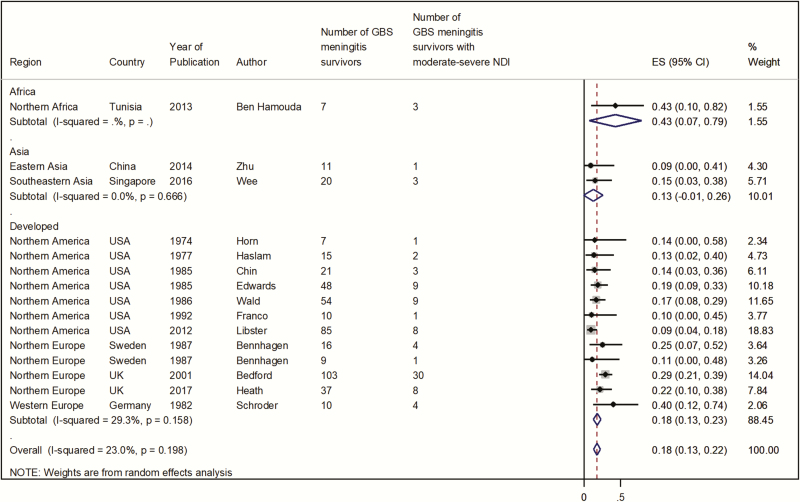
Infant group B *Streptococcus* meningitis survivors followed up for a median of ≥18 months with moderate to severe neurodevelopmental impairment. Abbreviations:

CI, confidence interval; ES, effect size; GBS, group B *Streptococcus*; NDI, neurodevelopmental impairment.

When followed up over a median of 18 months, 18% (95% CI, 13%–22%) (Figure 4) of GBS meningitis survivors had moderate to severe NDI. There were insufficient data for estimates by United Nations subregions, other than for developed countries, for which the estimate was also 18% (95% CI, 13%–23%), reflecting the fact that this was where most data were from.

Sensitivity meta-analyses were for risk of NDI in survivors of infant GBS meningitis by severity, length of follow-up, and NMR (per 1000 live births) context.

A series of 7 meta-analyses (meta-analyses 1–7) acted as sensitivity analyses to assess the variation of risk of NDI with as long follow-up as possible in different NMR settings. Supplementary Figure 1 demonstrates the number of studies included in each meta-analysis.

1. GBS meningitis survivors followed up for median of ≥18 months with any NDI: 32% (95% CI, 25%–38%) (Supplementary Figure 2) of infant GBS meningitis survivors had any NDI (mild, moderate, or severe) when followed up for a median of at least 18 months.
2. GBS meningitis survivors followed up for median of ≥18 months with moderate to severe NDI where NMR ≥5 per 1000: Percentage of GBS meningitis survivors with moderate to severe NDI was 18% (95% CI, 12%–24%) (Supplementary Figure 3) when followed up for a median of at least 18 months where NMR is ≥5 per 1000 live births.
3. GBS meningitis survivors followed up for median of ≥18 months with moderate to severe NDI where NMR <5 per 1000: At 18% (95% CI, 8%–28%) (Supplementary Figure 4), the percentage of GBS meningitis survivors with moderate to severe impairment where NMR is <5 per 1000 was very similar to results in settings where NMR is ≥5 per 1000 live births.
4. GBS meningitis survivors followed up for median of ≥6 months with any NDI: When followed up for a median of at least 6 months, 27% (95% CI, 20%–34%) GBS meningitis survivors were reported to have any NDI (Supplementary Figure 5).
5. GBS meningitis survivors followed up for median of ≥6 months with moderate to severe NDI: 15% (95% CI, 11%–20%) of GBS meningitis survivors were reported to have moderate to severe NDI at a median of at least 6 months of follow-up (Supplementary Figure 6).
6. GBS meningitis survivors followed up for median of ≥6 months with moderate to severe NDI where NMR is ≥5 per 1000: The percentage of GBS meningitis survivors with moderate to severe NDI was 15% (95% CI, 10%–20%) (Supplementary Figure 7) when followed up for a median of at least 6 months where NMR is ≥5 per 1000 live births.
7. GBS meningitis survivors followed up for median of ≥6 months with moderate to severe NDI where NMR is <5 per 1000: A slightly greater percentage of GBS meningitis survivors followed up for a median of at least 6 months in settings where NMR is <5 per 1000 had moderate to severe NDI, 18% (95% CI, 8%–28%) (Supplementary Figure 8), compared to settings where NMR is ≥5 per 1000.

Table 2 summarizes the results of the above meta-analyses, with more details in Supplementary Figures 2–8.

## DISCUSSION

GBS is an important contributor to NDI after infant meningitis, of which it is a leading cause. However, data are insufficient to assess its contribution to NDI after all infant GBS disease, which is important as there are many more cases of infant GBS sepsis compared to infant meningitis [27].

Our estimates of moderate to severe NDI following GBS meningitis in 18% (95% CI, 13%–22%) of survivors is consistent with the estimate of NDI after meningitis of all infectious etiologies, which is 23% (95% CI, 19%–26%) [7]. The slightly lower point estimate may be due to differences in the geographies covered. In our analysis, there were no data from low-income contexts, and data from only 3 middle-income contexts (China, Tunisia, and South Africa) and a higher proportion of data from developed countries compared to the previous work, which limits the generalizability of the NMR sensitivity analyses.

Improving the data are critical to direct public health interventions. Currently, and mainly in high-income contexts, early-onset cases (the first week after birth) of invasive GBS disease are reduced through intrapartum antibiotic chemoprophylaxis, but maternal vaccination may offer an alternative strategy in the future, with the potential to reduce the morbidity, as well as the mortality burden from GBS disease, and be more feasible in settings where a large proportion of deliveries occur at home. There were insufficient data to determine whether NDI in GBS meningitis survivors varies between different mortality contexts. For settings with an NMR <5 per 1000 live births and ≥5 per 1000 live births, the risk of moderate to severe NDI was 18% (8%–28%) and 18% (12%–24%), respectively, at median follow-up of at least 18 months. Whether there is a difference in very high-mortality settings (NMR >15/1000 live births) is unknown. However, as >90% of the world’s births are in low- or middle-income contexts, the number of cases are likely to be highest in these contexts [2].

Overall it is likely that we are underestimating NDI after invasive infant GBS disease, due to challenges in detection, particularly at the youngest ages. As expected, the prevalence of NDI increased with follow-up to 18 months compared to 6 months (18% [95% CI, 13%–22%] vs 15% [95% CI, 11%–20%]). While we acknowledge that these confidence intervals overlap considerably, the upward trend suggests that longer follow-up is needed for accurate case ascertainment. The range of assessment tools used for NDI diagnosis limits comparability, and demonstrates the need for standardization of methodologies, as well as validation of tools in different contexts. There were 44 different methods used in the studies in this review, which results in heterogeneity and lack of true comparability. This review is further limited by use of developmental screening tests by some studies, rather than diagnostic developmental assessments. While there is no universal gold standard for high-risk newborn follow-up, studies should ensure comprehensive neurodevelopment assessment of all domains, and of vision and hearing, and should align methods of assessment with contemporary studies. Neurodevelopment assessment tools, where not developed locally, should be translated, culturally adapted, and validated in the setting. NDI in GBS-associated preterm and neonatal encephalopathy survivors is also important to consider in estimating the total burden of GBS disease but was not investigated in this review.

**Figure 5.**
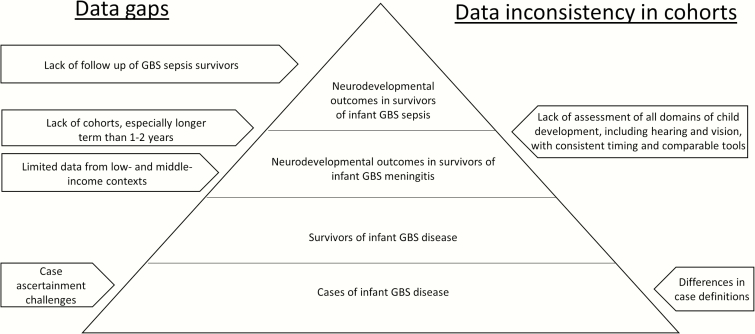
“Iceberg” of data available on neurodevelopmental outcomes after infant group B streptococcal (GBS) disease.

To improve the data, ideally, we should systematically investigate infants with signs of possible serious bacterial infection to ascertain a bacterial infectious etiology, and follow-up for longer periods of time to determine NDI outcomes, using standardized timing of neurodevelopmental assessment with tools validated in the context. To investigate the long-term impact of infant infection, particularly in terms of NDI, longer-term cohort studies are required (Figure 5). To avoid recruitment bias, study participants should be well described, including data on, for example, comorbidities, and gestational age and birthweight, which (if low) can contribute to NDI. Furthermore, while we have focused on physical NDI, a comprehensive assessment of all child development outcomes, including socioemotional and behavioral outcomes, would also improve understanding of the total burden of NDI after infant GBS disease [55, 56].

## CONCLUSIONS

GBS is a leading cause of infant meningitis, and almost one-fifth of GBS meningitis survivors experience moderate or severe NDI. There is an additional, as yet unquantified burden associated with other invasive infant disease, such as GBS sepsis. It is critical to look toward improving the health and well-being of survivors of infant GBS disease, and supporting their families, for whom there are financial, social, psychological, and emotional impacts. Prevention strategies (intrapartum antibiotic chemoprophylaxis) for early-onset invasive infant GBS disease are currently limited to developed countries, and only around the time of birth. Maternal GBS vaccination may be able to reduce the burden of GBS disease further, particularly GBS meningitis, which mainly presents as late-onset disease beyond 7 days of life (Table 3).

**Table 3.** Key Findings and Implications

What’s new about this? • The first systematic review of NDI findings after GBS and, despite 3 decades of focus on GBS disease in infants, data were insufficient to assess NDI outcomes after GBS sepsis. Only 18 studies met inclusion criteria for follow-up after GBS meningitis.
What was the main finding? • Eighteen percent (95% CI, 13%–22%) of survivors of infant GBS meningitis have moderate or severe NDI at a median follow-up time of >18 months.
How can the data be improved? • Cohort studies with adequate follow-up (>18 months) using standardized assessment tools, validated locally, and times of clinical review, particularly in resource-poor settings.
What does it mean for policy and programs? • Lack of data limits our assessment of the burden of NDI after invasive infant GBS disease, which may be a considerable burden and increases with improvements in survival.

Abbreviations: CI, confidence interval; GBS, group B Streptococcus; NDI, neurodevelopmental impairment; NMR, neonatal mortality rate per 1000 live births.

## Supplementary Data

Supplementary materials are available at *Clinical Infectious Diseases* online. Consisting of data provided by the authors to benefit the reader, the posted materials are not copyedited and are the sole responsibility of the authors, so questions or comments should be addressed to the corresponding author.

## Supplementary Material

supplement-material

## Notes

***Author contributions.*** The concept of the estimates and the technical oversight of the series was led by J. E. L. and A. C. S.. The reviews, analyses, and first draft of the paper were undertaken by M. K. L. with N. R. and J. E. L. Other specific contributions were made by A. C. S., C. J. T., and Z. D. The GBS Estimates Expert Advisory Group (C. J. B., L. B., C. C., M. G. G., P. T. H., M. I., K. L. D., S. A. M., C. E. R., S. K. S., S. S., A. S.-t. M., J. V.) contributed to the conceptual process throughout, notably on the disease schema and data inputs. The GBS NDI Investigator Group (C. O. S., F. N., H. B. H., H. S., K. G., S. L., T. L., H. R., and C. T.) input data for the analyses. All the authors reviewed and gave input to the manuscript.

***Acknowledgments.*** We thank the Meningitis Research Foundation as funder of unpublished data; the GBS Expert Advisory Group and GBS NDI Investigator Group (listed below); Claudia da Silva for administrative assistance; and Alegria Perez for coordinating author signatures.

***Financial support.*** This supplement was supported by a grant to the London School of Hygiene & Tropical Medicine from the Bill & Melinda Gates Foundation (reference number OPP1131158).

***Supplement sponsorship.*** This article appears as part of the supplement “The Burden of Group B *Streptococcus* Worldwide for Pregnant Women, Stillbirths, and Children,” sponsored by the Bill & Melinda Gates Foundation and coordinated by the London School of Hygiene & Tropical Medicine.

***Potential conflicts of interest.*** Many contributors to this supplement have received funding for their research from foundations, especially the Bill & Melinda Gates Foundation, and several from Wellcome Trust, Medical Research Council UK, the Thrasher Foundation, the Meningitis Research Foundation, and one individual from the US National Institutes of Health. Members of the Expert Advisory Group received reimbursement for travel expenses to attend working meetings related to this series. A. S.-t. M. works for the Bill & Melinda Gates Foundation. C. J. B. has served as a member of the Presidential Advisory Committee for Seqirus Inc and of the CureVac Inc Scientific Advisory Committee, as well as undertaken consultancy work for Pfizer Inc. C. C. has received institutional compensation from Novartis for conducting GBS studies. P. T. H. has been a consultant to Novartis and Pfizer on GBS vaccines but received no funding for these activities. M. I. has undertaken sponsored research from Pfizer on pneumococcal disease in adults and from Belpharma Eumedica (Belgium) on temocillin antimicrobial susceptibility in Enterobacteriaceae. K. L. D. has received funding by the Bill & Melinda Gates Foundation to work on research on GBS serocorrelates of protection to inform vaccine trials, and travel expenses from Pfizer to attend a meeting on an investigator-led project on GBS. S. A. M. has collaborated on GBS grants funded by GlaxoSmithKline and by Pfizer and received personal fees for being member of its advisory committee; he has also collaborated on a GBS grant funded by Minervax. All other authors report no potential conflicts of interest. All authors have submitted the ICMJE Form for Disclosure of Potential Conflicts of Interest. Conflicts that the editors consider relevant to the content of the manuscript have been disclosed.
