# Supplementary material for: Neurodevelopmental Impairment in Children After Group B Streptococcal Disease Worldwide: Systematic Review and Meta-analyses
Source: Clin Infect Dis. 2017 Nov 6;65(Suppl 2):S190–9. doi: 10.1093/cid/cix663 (PMC5848372; doi:10.1093/cid/cix663)
Supplement: supplement-material [file cix663_suppl_supplement-material.pdf]

The burden of Group B *Streptococcus* worldwide for pregnant women, stillbirths and children

Paper 10: Neurodevelopmental impairment in children after Group B *Streptococcus* disease worldwide: systematic review and meta-analyses

Supplementary information

Contents

Table S1a: Group B *Streptococcus* syndrome definitions 3

Table S1b: Impairment domain severity definitions and disability weights 4

Table S2: World Health Organization International Classification of Functioning, Disability and Health definitions [7] 7

Table S3: Search terms 8

Table S4: Inclusion and exclusion criteria 9

Table S5: Neurodevelopmental impairment in survivors of infant Group B *Streptococcus* sepsis 10

Table S6: Neurodevelopment Assessments 11

Figure S1: Organogram of meta-analyses performed with number of studies included in each meta-analysis 13

Figure S2: Meta-analysis A. Infant Group B *Streptococcus* meningitis survivors followed up for median of ≥18 months with any NDI 14

Figure S3: Meta-analysis B. Infant Group B *Streptococcus* meningitis survivors followed up for median of ≥18 months with moderate-severe NDI where NMR ≥5 / 1000 15

Figure S4: Meta-analysis C. Infant Group B *Streptococcus* meningitis survivors followed up for median of ≥18 months with moderate-severe NDI where neonatal mortality rate (NMR) <5 / 1000 16

Figure S5: Meta-analysis D. Infant Group B *Streptococcus* meningitis survivors followed up for median of ≥6 months with any NDI 17

Figure S6: Meta-analysis E. Infant Group B *Streptococcus* meningitis survivors followed up for median of ≥6 months with moderate-severe NDI 18

Figure S7: Meta-analysis F. Infant Group B *Streptococcus* meningitis survivors followed up for median of ≥6 months with moderate-severe NDI where NMR ≥5 / 1000 19

Figure S8: Meta-analysis G. Infant Group B *Streptococcus* meningitis survivors Group B *Streptococcus* meningitis survivors followed up for median of ≥6 months with moderate-severe NDI where NMR <5 / 1000 20

References 21

Table S1a: Group B *Streptococcus* syndrome definitions

GBS Syndrome	Definition	ICD-10 code
**Possible serious bacterial infection (pSBI)**	Any one of the following: a history of difficulty feeding, history of convulsions, movement only when stimulated, respiratory rate of 60 breaths per min or more, severe chest indrawing, temperature ≥ 37.5°C or ≤35.5°C [1]	P35-P39
**Sepsis**	Clinical signs of pSBI *and* GBS-positive blood culture or PCR or latex agglutination	P36.0
**Meningitis**	Clinical signs of pSBI *and* [GBS-positive CSF culture or PCR or latex agglutination *or* (GBS-positive blood culture or PCR or latex agglutination *and* CSF leucocyte count of >$20x10^6$ /l)] [2]	G00.0-G00.9
**Pneumonia**	Clinical signs (fast breathing, in-drawing) *and* informed by chest x-ray *and* GBS-positive blood culture or PCR or latex agglutination [3]	P23.0-P29, J15.3

Table S1b: Impairment domain severity definitions and disability weights

Impairment (ICD10 code) [4]	Domain	Definition used in this paper (modified from GBD2013 and Nature supplement) [5, 6]	Global Burden of Disease Study 2013 (GBD2013) lay definition	GBD2013 Disability Weight
**Motor**<br><br>**(G80, G81.1, G82.1, G83.0-G83.3, G24.9, G25.3, G25.5, G25.9, F82, R26-R27)**	Mild motor	Difficulty in everyday motor activities but able to move around without help.	Difficulty in moving around but is able to walk without help.	0.010 (0.005-0.019)
	Moderate motor	Difficulty in holding implements, dressing and sitting upright. Able to move around without help.	Difficulty in moving around, and difficulty in lifting and holding objects, dressing and sitting upright, but is able to walk without help.	0.061 (0.040-0.089)
	Severe motor	Inability to walk and absence of functional use of hands.	Unable to move around without help, and not able to lift or hold objects, get dressed or sit upright.	0.402 (0.268-0.545)
**Intellectual**<br><br>**(F70-F79, F80.0, F81.0, F81.2, F81.8, F81.9, F06.7)**	Mild intellectual	Cognitive Z-score -1 to -2 SD for test (DQ 70-84)	Low intelligence and is slow in learning at school.	0.043 (0.026, 0.064)
	Moderate intellectual	Cognitive Z-score -2 to -3 SD for test (DQ 55-69)	Low intelligence, and is slow in learning to speak and to do even simple tasks.	0.100 (0.066, 0.142)
	Severe intellectual	Cognitive Z-score ≤3 for test (DQ <55)	Very low intelligence and cannot speak more than a few words, needs constant supervision and help with most daily activities, and can do only the simplest tasks.	0.160 (0.107, 0.226)
	Profound intellectual	Cognitive Z-score ≤3 for test (DQ <55)	Very low intelligence, has almost no language, and does not understand even the most basic requests or instructions. Requires constant supervision and help for all activities.	0.200 (0.133, 0.283)
**Motor and intellectual**	Mild motor and intellectual	Mild motor and mild intellectual	Some difficulty in moving around but is able to walk without help. Slow in learning at school.	0.031 (0.018, 0.050)

	Moderate motor and intellectual	Moderate motor and intellectual impairment	Some difficulty in moving around, holding objects, dressing and sitting upright, but can walk without help. Low intelligence and is slow in learning to speak and to do simple tasks.	0.203 (0.134, 0.290)
	Severe motor and intellectual	Severe motor and severe intellectual	Cannot move around without help, and cannot lift or hold objects, get dressed or sit upright. Very low intelligence, speaks few words, and needs constant supervision and help with all daily activities.	0.542 (0.374, 0.702)
**Visual**<br><br>**(H47, H48-H51, H53.0, H53.2, H53.4, H54.4-H54.7)**	Mild visual	Visual acuity in best eye <6/12 but better or equal to 6/185 or corresponding visual field loss	Some difficulty with distance vision, for example reading signs, but no other problems with eyesight.	0.003 (0.001-0.007)
	Moderate visual	Visual acuity in best eye between 6/18 and 6/60, or corresponding visual field loss	Vision problems that make it difficult to recognize faces or objects across a room.	0.031 (0.019-0.049)
	Severe visual	Visual acuity in best eye between 6/60 and 3/60, or corresponding visual field loss	Severe vision loss, which causes difficulty in daily activities, some emotional impact (for example worry), and some difficulty going outside the home without assistance.	0.184 (0.125-0.258)
	Blind	Visual acuity in best eye <3/60, or corresponding visual field loss	Completely blind, which causes great difficulty in some daily activities, worry and anxiety, and great difficulty going outside the home without assistance.	0.187 (0.124-0.260)
**Hearing**<br><br>**(H90.3-H90.7)**	Mild hearing	Audiometric hearing threshold level 26-30 decibel	Great difficulty hearing and understanding another person talking in a noisy place (for example, on an urban street).	0.010 (0.004, 0.019)
	Moderate hearing	Audiometric hearing threshold level 31-64.9 decibel	Unable to hear and understand another person talking in a noisy place (for example, on an urban street), and has difficulty hearing another person talking even in a quiet place or on the phone.	0.027 (0.015, 0.042)
	Severe hearing	Audiometric hearing threshold level 65-89.9 decibel	Unable to hear and understand another person talking, even in a quiet place, and unable to take	0.158 (0.105, 0.227)

			part in a phone conversation. Difficulties with communicating and relating to others cause emotional impact at times (for example worry or depression).	
	Profound hearing	Audiometric hearing threshold level >90 decibel	Unable to hear and understand another person talking, even in a quiet place, is unable to take part in a phone conversation, and has great difficulty hearing anything in any other situation. Difficulties with communicating and relating to others often cause worry, depression or loneliness.	0.204 (0.134, 0.288)
	Complete hearing loss	Cannot hear at all in any situation including even the loudest sounds	Cannot hear at all in any situation, including even the loudest sounds, and cannot communicate verbally or use a phone. Difficulties with communicating and relating to others often cause worry, depression or loneliness.	0.215 (0.144, 0.307

Table S2: World Health Organization International Classification of Functioning, Disability and Health definitions [7]

Term	Definition
**Impairment**	problems in body function and structure such as significant deviation or loss
**Activity limitation**	a difficulty encountered by an individual in executing a task or action
**Participation restriction**	a problem experienced by an individual in involvement in life situations
**Disability**	an umbrella term, covering impairment, activity limitation, and participation restriction

Table S3: Search terms

Brain Damage, Chronic[MeSH Terms]	Epilepsy	sequel*
Cerebral palsy [MeSH Terms]	Seizures [MeSH Terms]	outcome
Cerebral palsy	Seizures	Development
Cerebral AND palsy	Vision Disorders [MeSH Terms]	Neurodevelopment*
Disabled Children [MeSH Terms]	Blindness	Neuro- development*
Mental Disorders Diagnosed in Childhood [MeSH Terms]	Blind	Death
Mental Retardation [MeSH Terms]	Hearing Loss, Sensorineural [MeSH Terms]	Mortality
Learning Disorders [MeSH Terms]	Hearing Loss, Sensorineural [MeSH Terms]	Case AND Fatality AND rate
Communication Disorders [MeSH Terms]	Hearing Loss	Fatality
Developmental Disabilities [MeSH Terms]	Hearing AND loss	Death [MeSH Terms]
Cognitive AND impairment	Deafness [MeSH Terms]	Mortality [MeSH Terms]
Motor AND impairment	Prognosis [MeSH Terms]	Morbidity [MeSH Terms]
Epilepsy [MeSH Terms]	Impairment	Case fatality rate [MeSH Terms]

AND

Streptococcus
Streptococcal
Streptococci AND (Group AND B) or Agalactiae
Streptococcus Agalactiae [MeSH Terms]

Table S4: Inclusion and exclusion criteria

	Inclusion criteria	Exclusion criteria
**Population**	Invasive GBS disease<br>Index case <90 days of life	Non-representative sample (e.g. all preterm infants, all stroke survivors)
**Case definition**	Neurodevelopmental outcomes reported at ≥6 months	Cases not pathogen-specific
**Laboratory**	GBS confirmed by blood / CSF culture or PCR or latex agglutination	
**Search**	No language or date restrictions	
**Article type**		Review articles, case reports

Table S5: Neurodevelopmental impairment in survivors of infant Group B *Streptococcus* sepsis

UN region	UN sub-region	Country	Author	Publication year	Median year data collection	NMR (per 1000 live births)	No. of GBS sepsis survivors	No. of GBS sepsis survivors with any NDI (%)	No. of GBS sepsis survivors with mod-sev NDI (%)
Africa	Southern Africa	South Africa	Dangor	(unpublished)	2014	11	70	13 (18.6)	5 (7.1)
		USA	Horn	1974	unknown	12	4	0 (0)	0 (0)
Europe	Northern Europe	UK	Heath	(unpublished)	2015.5	2	61	5 (8.2)	1 (1.6)
		Denmark	Carstensen	1985	1980.5	6	36	1 (2.8)	0 (0)
	Western Europe	Germany	Schroder	1982	1975.5	9	8	2 (25)	1 (12.5)

NDI=Neurodevelopmental impairment, NMR=neonatal mortality rate, mod-sev=moderate to severe

Table S6: Neurodevelopment Assessments

UN Region	Author	No. of assess ments	Age(s) of child at time of assessment (years)	Cognitive	Motor	Language	Vision	Hearing	Socio-emotional / Behavioural
Africa	Dangor	3	0.3, 0.5, 1.0	-	DDST II, Paed. exam	DDST II, Paed. exam	-	-	DDST II
	Ben Hamouda	1	Range: 1.0-9.0	Paed. exam	Paed. exam	Paed. exam	VER	BAER	Paed. exam
Americas	Libster	1	Range: 3.0-12.0	WIAT II	MSEL, CDI Paed. Exam	MSEL, CDI	MSEL, SVAC	Audiometr y	PEDS, CDI
	Franco	19	0.3, 0.5, 0.8, 1, 1.5, 2.0, 3.0, 4.0, 5.0, 6.0, 7.0, 8.0, 9.0, 10.0, 11.0, 12.0, 13.0, 14.0	SBIS, CIIS WISC, WPPSI	Paed. Exam	(Method not described)	(Method not described)	(Method not described)	-
	Wald	1	Range: 3.0-18.0	WPPSI, WISC, MSCA, WRAT, SAT	GPT, BVMI	-	BVMI	Audiometr y	CBC, VABS
	Chin	5	0.5, 0.8, 1.0, 1.5 then Range: 5.0-7.0	BSID, WPPSI, WISC	BSID, Paed. exam	BSID	Fundoscopy , refraction	SFA	-
	Edwards	1	Range: 3.3-9.0	BSID, MCDI, WJPEB	BSID, MCDI	BSID, PLS, PPVT, EOPT, CELI, ITPA, TELD	Ophthalm. exam	ENT exam	Psych. exam
	Haslam	1	Range: 0.6-6.9	BSID, SBIS, WISC, psych. exam	BSID, Paed. exam	BSID	(Method not described)	PTA, BOA	Psych. exam
	Horn	1	Range: 1.1-3.5	-	DDST II, Paed. Exam	DDST II, Paed. exam	-	-	DDST II
	Baker	1	Range: 0.5-2.0	Q’aire to paed.	Q’aire to paed.	-	-	-	-
Asia	Wee	4	0.5, 1.0, 2.0, 5.0	Paed. exam	Paed. Exam	Paed. Exam	(Method not described)	ENT exam	-
	Zhu	1	Range: 0.8-2.0	-	Q’aire to	-	-	-	-

					parents				
Europe	Bedford	1	5.0	Q'air e to parents	Q'aire to parents	Q'aire to parents	Q'aire to parents	Q'aire to parents	Q'aire to parents
	Bennhagen	1	Range: 1.3-2.3	GP/paed. exam	GP/paed. Exam	GP/paed. exam	(Method not described)	(Method not described)	-
	Carstensen	Not reported	Range :0.3-5.0	Paed. exam	Paed. Exam	Paed. Exam	(Method not described)	(Method not described)	-
	Heath	1	Range: 2.0-4.0	BSID	BSID	BSID	Health assessment	Health assessme nt	-
	Schroder	1	Range: 0.8-13.0	-	DDST II	MFDD, DDST II	(Method not described)	Audiometr y	DDST II

BA ER – Brainstem auditory evoked response, BVMI – Beery Test of Visual-Motor Integration [8], BOA – Behavioural Observation Audiometry [9], BSID – Bayley's Scales of Infant Development [10], CBC – Child Behaviour Checklist [11], CDI – Child Development Inventory [12], CELI - Carrow Elicited Language Inventory [13], CIIS – Cattell Infant Intelligence Scale [14], DDST II – Denver Developmental Screening Test II [15], ENT exam – otolaryngologist examination, EOWPT - Expressive One Word Picture Vocabulary Test [16], GP exam - Neurological / developmental examination by General Practitioner, GPT - Grooved Pegboard Test [17], ITPA - Illinois Test of Psycholinguistic Abilities [18], MCDI - Minnesota Child Development Inventory [19], MFDD - Munich Functional Development Diagnostic [20], MSCA - McCarthy Scale of Children's Abilities [21], MSEL - Mullen's Scale of Early Learning [22] , Ophthalm. Exam – ophthalmologist examination, Paed. Exam – Neurological / developmental examination by paediatrician, PEDS – Parent Evaluation of Development Status [23], PLS – Preschool Language Scale [24], PPVT - Peabody Picture Vocabulary Test [25], PTA – Pure Tone Audiometry [26], Psych. Exam – psychologist examination, Q'aire – Questionnaire, SAT – school achievement test, SBIS – Stanford-Binet Intelligence Scales [27], SFA – Sound Field Audiometry [28], SVAC - Snellen Visual Acuity Chart [29], TELD - Test of Early Language Development [30], VABS – Vineland Adaptive Behaviour Scales [31], VER – Visual Evoked Response, WIAT II – Weshler Individual Achievement Test II [32], WISC – Weschler Intelligence Scale for Children [33], WJPEB - Woodcock-Johnson Psycho-Educational Battery [34], WPPSI – Weschler Preschool and Primary Scale [35], WRAT – Wide Range Achievement Test [36]

Figure S1: Organogram of meta-analyses performed with number of studies included in each meta-analysis

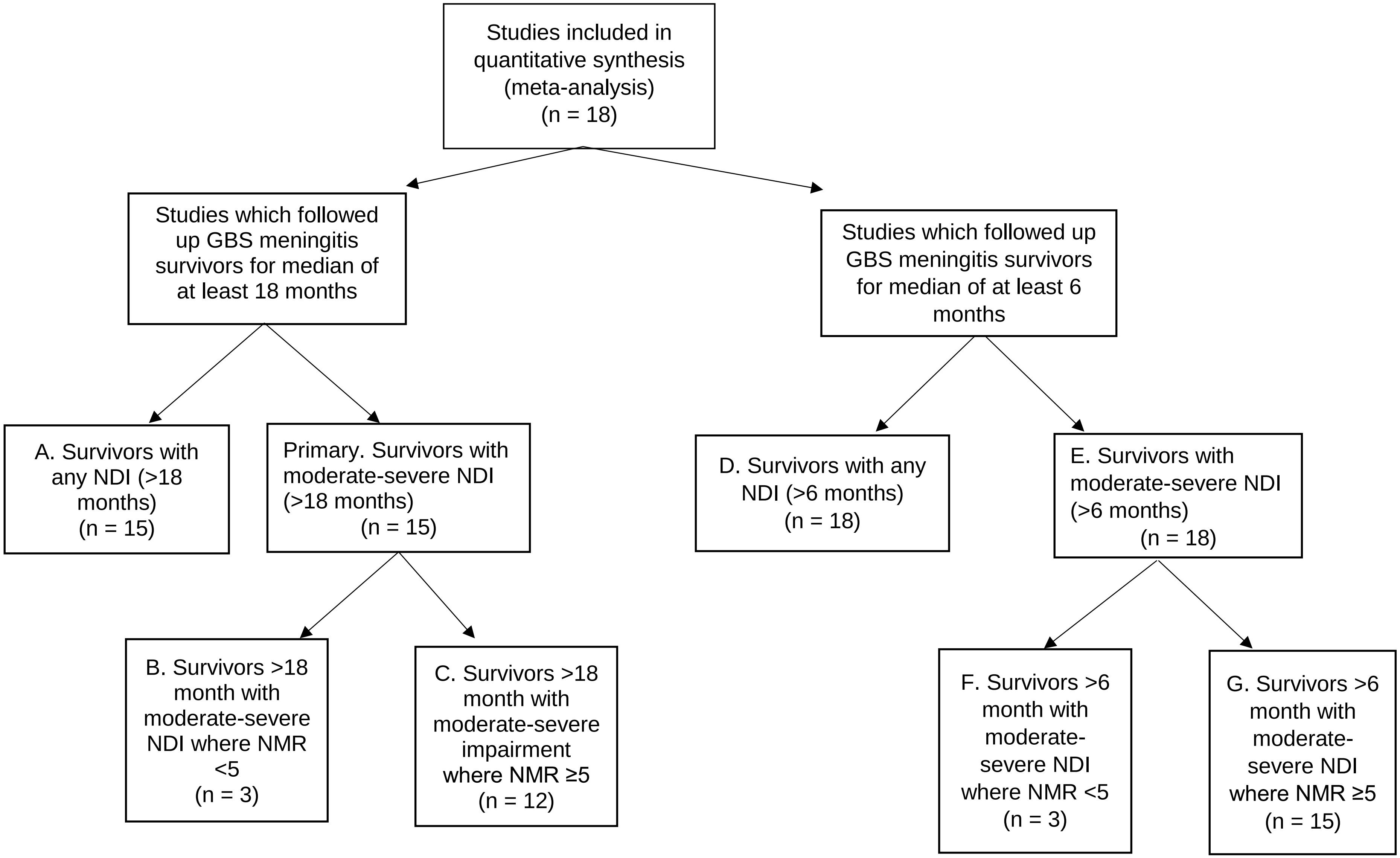

NDI=Neurodevelopmental impairment, NMR=neonatal mortality rate (per 1000 live births)

Figure S2: Meta-analysis A. Infant Group B *Streptococcus* meningitis survivors followed up for median of ≥18 months with any NDI

Region	Country	Year of Publication	Author	Number of GBS meningitis survivors	No. of survivors with NDI	ES (95% CI)	% Weight
Africa							
Northern Africa	Tunisia	2013	Ben Hamouda	7	4	0.57 (0.18, 0.90)	2.74
Subtotal (I-squared = .%, p = .)						0.57 (0.21, 0.93)	2.74
.							
Asia							
Eastern Asia	China	2014	Zhu	11	4	0.36 (0.11, 0.69)	3.84
Southeastern Asia	Singapore	2016	Wee	20	4	0.20 (0.06, 0.44)	6.99
Subtotal (I-squared = 0.0%, p = 0.356)						0.25 (0.09, 0.41)	10.83
.							
Developed							
Northern America	USA	1974	Horn	7	2	0.29 (0.04, 0.71)	3.05
Northern America	USA	1977	Haslam	15	4	0.27 (0.08, 0.55)	5.24
Northern America	USA	1985	Chin	21	6	0.29 (0.11, 0.52)	6.37
Northern America	USA	1985	Edwards	48	19	0.40 (0.26, 0.55)	9.34
Northern America	USA	1986	Wald	54	14	0.26 (0.15, 0.40)	10.71
Northern America	USA	1992	Franco	10	6	0.60 (0.26, 0.88)	3.52
Northern America	USA	2012	Libster	85	19	0.22 (0.14, 0.33)	12.87
Northern Europe	Sweden	1987	Bennhagen	16	4	0.25 (0.07, 0.52)	5.59
Northern Europe	Sweden	1987	Bennhagen	9	1	0.11 (0.00, 0.48)	5.14
Northern Europe	UK	2001	Bedford	103	48	0.47 (0.37, 0.57)	12.38
Northern Europe	UK	2017	Heath	37	11	0.30 (0.16, 0.47)	8.71
Western Europe	Germany	1982	Schroder	10	4	0.40 (0.12, 0.74)	3.52
Subtotal (I-squared = 48.7%, p = 0.029)						0.32 (0.24, 0.39)	86.43
.							
Overall (I-squared = 43.8%, p = 0.035)						0.32 (0.25, 0.38)	100.00

NOTE: Weights are from random effects analysis

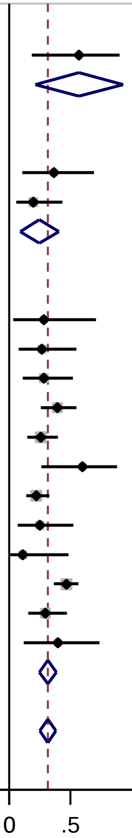

ES=Effect size, NDI=Neurodevelopmental impairment

Figure S3: Meta-analysis B. Infant Group B *Streptococcus* meningitis survivors followed up for median of ≥18 months with moderate-severe NDI where NMR ≥5 / 1000

Region	Country	Median year of data collection	Author	Number of GBS meningitis survivors	Number of GBS meningitis survivors with moderate-severe NDI	Neonatal mortality rate	ES (95% CI)	% Weight
Africa								
Northern Africa	Tunisia	2003	Ben Hamouda	7	3	15	0.43 (0.10, 0.82)	2.28
Subtotal (I-squared = .%, p = .)							0.43 (0.07, 0.79)	2.28
.								
Asia								
Eastern Asia	China	2009	Zhu	11	1	9	0.09 (0.00, 0.41)	5.91
Subtotal (I-squared = .%, p = .)							0.09 (-0.11, 0.30)	5.91
.								
Developed								
Northern America	USA		Horn	7	1	12	0.14 (0.00, 0.58)	3.38
Northern America	USA	1971	Haslam	15	2	14	0.13 (0.02, 0.40)	6.44
Northern America	USA	1973	Wald	54	9	13	0.17 (0.08, 0.29)	13.60
Northern America	USA	1975	Franco	10	1	12	0.10 (0.00, 0.45)	5.25
Northern America	USA	1976.5	Edwards	48	9	10	0.19 (0.09, 0.33)	12.25
Northern America	USA	1978	Chin	21	3	9	0.14 (0.03, 0.36)	8.05
Northern America	USA	2002	Libster	85	8	5	0.09 (0.04, 0.18)	19.14
Northern Europe	Sweden	1976	Bennhagen	16	4	6	0.25 (0.07, 0.52)	5.09
Northern Europe	UK	1986	Bedford	103	30	5	0.29 (0.21, 0.39)	15.61
Western Europe	Germany	1975.5	Schroder	10	4	9	0.40 (0.12, 0.74)	3.00
Subtotal (I-squared = 39.7%, p = 0.093)							0.18 (0.12, 0.24)	91.81
.								
Overall (I-squared = 37.1%, p = 0.094)							0.18 (0.12, 0.24)	100.00

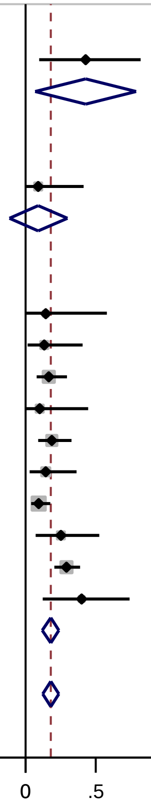

NOTE: Weights are from random effects analysis

Note: Where median year of data collection unknown (Horn, 1974) [37], neonatal mortality rate at year of publication used. ES=Effect size, NDI=Neurodevelopmental impairment

Figure S4: Meta-analysis C. Infant Group B *Streptococcus* meningitis survivors followed up for median of ≥18 months with moderate-severe NDI where neonatal mortality rate (NMR) <5 / 1000

Region	Country	Median year of data collection	Author	Number of GBS meningitis survivors	Number of GBS meningitis survivors with moderate-severe NDI	Neonatal mortality rate	ES (95% CI)	% Weight
Asia								
Southeastern Asia	Singapore	2005.5	Wee	20	3	1	0.15 (0.03, 0.38)	33.16
Subtotal (I-squared = .%, p = .)							0.15 (-0.02, 0.32)	33.16
.								
Developed								
Northern Europe	Sweden	1983	Bennhagen	9	1	4	0.11 (0.00, 0.48)	17.34
Northern Europe	UK	2015.5	Heath	37	8	2	0.22 (0.10, 0.38)	49.50
Subtotal (I-squared = 0.0%, p = 0.460)							0.19 (0.07, 0.31)	66.84
.								
Overall (I-squared = 0.0%, p = 0.713)							0.18 (0.08, 0.28)	100.00

NOTE: Weights are from random effects analysis

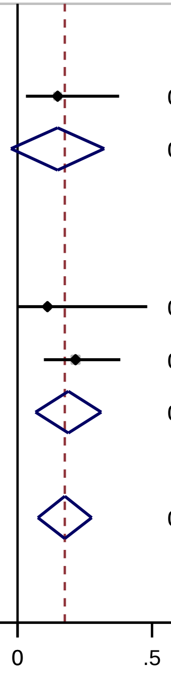

ES=Effect size, NDI=Neurodevelopmental impairment

Figure S5: Meta-analysis D. Infant Group B *Streptococcus* meningitis survivors followed up for median of ≥6 months with any NDI

Region	Country	Year of Publication	Author	Number of GBS meningitis survivors	No. of survivors with NDI	ES (95% CI)	% Weight
Africa							
Northern Africa	Tunisia	2013	Ben Hamouda	7	4	0.57 (0.18, 0.90)	2.86
Southern Africa	South Africa	2016	Dangor	30	3	0.10 (0.02, 0.27)	7.33
Subtotal (I-squared = 83.2%, p = 0.015)						0.30 (-0.15, 0.76)	10.20
.							
Asia							
Eastern Asia	China	2014	Zhu	11	4	0.36 (0.11, 0.69)	3.74
Southeastern Asia	Singapore	2016	Wee	20	4	0.20 (0.06, 0.44)	5.69
Subtotal (I-squared = 0.0%, p = 0.356)						0.25 (0.09, 0.41)	9.43
.							
Developed							
Northern America	USA	1973	Baker	23	1	0.04 (0.00, 0.22)	7.65
Northern America	USA	1974	Horn	7	2	0.29 (0.04, 0.71)	3.12
Northern America	USA	1977	Haslam	15	4	0.27 (0.08, 0.55)	4.69
Northern America	USA	1985	Chin	21	6	0.29 (0.11, 0.52)	5.36
Northern America	USA	1985	Edwards	48	19	0.40 (0.26, 0.55)	6.77
Northern America	USA	1986	Wald	54	14	0.26 (0.15, 0.40)	7.30
Northern America	USA	1992	Franco	10	6	0.60 (0.26, 0.88)	3.49
Northern America	USA	2012	Libster	85	19	0.22 (0.14, 0.33)	8.02
Northern Europe	Denmark	1985	Carstensen	26	4	0.15 (0.04, 0.35)	6.58
Northern Europe	Sweden	1987	Bennhagen	16	4	0.25 (0.07, 0.52)	4.91
Northern Europe	Sweden	1987	Bennhagen	9	1	0.11 (0.00, 0.48)	4.63
Northern Europe	UK	2001	Bedford	103	48	0.47 (0.37, 0.57)	7.87
Northern Europe	UK	2017	Heath	37	11	0.30 (0.16, 0.47)	6.50
Western Europe	Germany	1982	Schroder	10	4	0.40 (0.12, 0.74)	3.49
Subtotal (I-squared = 70.7%, p = 0.000)						0.28 (0.19, 0.36)	80.37
.							
Overall (I-squared = 69.0%, p = 0.000)						0.27 (0.20, 0.34)	100.00

NOTE: Weights are from random effects analysis

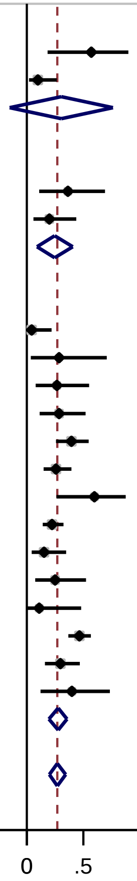

Note: Where study has not yet been published, 'Year of Publication' refers to final year of data collection
ES=Effect size, NDI=Neurodevelopmental impairment

Figure S6: Meta-analysis E. Infant Group B *Streptococcus* meningitis survivors followed up for median of ≥6 months with moderate-severe NDI

Region	Country	Year of Publication	Author	Number of GBS meningitis survivors	Number of GBS meningitis survivors with moderate-severe NDI	ES (95% CI)	% Weight
Africa							
Northern Africa	Tunisia	2013	Ben Hamouda	7	3	0.43 (0.10, 0.82)	1.33
Southern Africa	South Africa	2016	Dangor	30	2	0.07 (0.01, 0.22)	8.73
Subtotal (I-squared = 72.2%, p = 0.058)						0.21 (-0.14, 0.55)	10.06
.							
Asia							
Eastern Asia	China	2014	Zhu	11	1	0.09 (0.00, 0.41)	3.54
Southeastern Asia	Singapore	2016	Wee	20	3	0.15 (0.03, 0.38)	4.62
Subtotal (I-squared = 0.0%, p = 0.666)						0.13 (-0.01, 0.26)	8.16
.							
Developed							
Northern America	USA	1973	Baker	23	1	0.04 (0.00, 0.22)	8.48
Northern America	USA	1974	Horn	7	1	0.14 (0.00, 0.58)	1.99
Northern America	USA	1977	Haslam	15	2	0.13 (0.02, 0.40)	3.88
Northern America	USA	1985	Chin	21	3	0.14 (0.03, 0.36)	4.91
Northern America	USA	1985	Edwards	48	9	0.19 (0.09, 0.33)	7.73
Northern America	USA	1986	Wald	54	9	0.17 (0.08, 0.29)	8.68
Northern America	USA	1992	Franco	10	1	0.10 (0.00, 0.45)	3.13
Northern America	USA	2012	Libster	85	8	0.09 (0.04, 0.18)	12.81
Northern Europe	Denmark	1985	Carstensen	26	3	0.12 (0.02, 0.30)	6.35
Northern Europe	Sweden	1987	Bennhagen	16	4	0.25 (0.07, 0.52)	3.03
Northern Europe	Sweden	1987	Bennhagen	9	1	0.11 (0.00, 0.48)	2.73
Northern Europe	UK	2001	Bedford	103	30	0.29 (0.21, 0.39)	10.14
Northern Europe	UK	2017	Heath	37	8	0.22 (0.10, 0.38)	6.15
Western Europe	Germany	1982	Schroder	10	4	0.40 (0.12, 0.74)	1.76
Subtotal (I-squared = 36.7%, p = 0.083)						0.16 (0.11, 0.21)	81.78
.							
Overall (I-squared = 33.5%, p = 0.083)						0.15 (0.11, 0.20)	100.00

NOTE: Weights are from random effects analysis

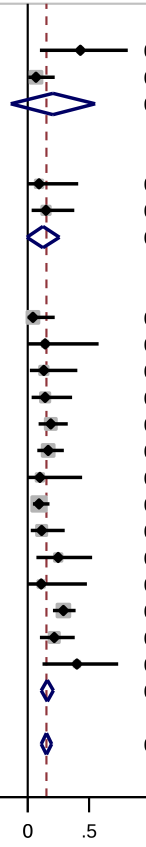

Note: Where study has not yet been published, 'Year of Publication' refers to final year of data collection
ES=Effect size, NDI=Neurodevelopmental impairment

Figure S7: Meta-analysis F. Infant Group B *Streptococcus* meningitis survivors followed up for median of ≥6 months with moderate-severe NDI where NMR ≥5 / 1000

Region	Country	Median year of data collection	Author	Number of GBS meningitis survivors	Number of GBS meningitis survivors with moderate-severe NDI	Neonatal mortality rate	ES (95% CI)	% Weight
Africa								
Northern Africa	Tunisia	2003	Ben Hamouda	7	3	15	0.43 (0.10, 0.82)	1.74
Southern Africa	South Africa	2014	Dangor	30	2	11	0.07 (0.01, 0.22)	9.88
Subtotal (I-squared = 72.2%, p = 0.058)							0.21 (-0.14, 0.55)	11.62
.								
Asia								
Eastern Asia	China	2009	Zhu	11	1	9	0.09 (0.00, 0.41)	4.42
Subtotal (I-squared = .%, p = .)							0.09 (-0.11, 0.30)	4.42
.								
Developed								
Northern America	USA		Horn	7	1	12	0.14 (0.00, 0.58)	2.56
Northern America	USA	1971	Baker	23	1	14	0.04 (0.00, 0.22)	9.64
Northern America	USA	1971	Haslam	15	2	14	0.13 (0.02, 0.40)	4.81
Northern America	USA	1973	Wald	54	9	13	0.17 (0.08, 0.29)	9.83
Northern America	USA	1975	Franco	10	1	12	0.10 (0.00, 0.45)	3.94
Northern America	USA	1976.5	Edwards	48	9	10	0.19 (0.09, 0.33)	8.91
Northern America	USA	1978	Chin	21	3	9	0.14 (0.03, 0.36)	5.96
Northern America	USA	2002	Libster	85	8	5	0.09 (0.04, 0.18)	13.51
Northern Europe	Denmark	1980.5	Carstensen	26	3	6	0.12 (0.02, 0.30)	7.51
Northern Europe	Sweden	1976	Bennhagen	16	4	6	0.25 (0.07, 0.52)	3.82
Northern Europe	UK	1986	Bedford	103	30	5	0.29 (0.21, 0.39)	11.19
Western Europe	Germany	1975.5	Schroder	10	4	9	0.40 (0.12, 0.74)	2.27
Subtotal (I-squared = 44.0%, p = 0.050)							0.16 (0.10, 0.21)	83.96
.								
Overall (I-squared = 42.9%, p = 0.040)							0.15 (0.10, 0.20)	100.00

NOTE: Weights are from random effects analysis

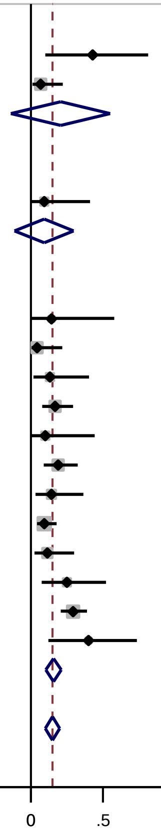

Note: Where median year of data collection unknown (Horn, 1974) [37], neonatal mortality rate at year of publication used.
ES=Effect size, NDI=Neurodevelopmental impairment

Figure S8: Meta-analysis G. Infant Group B *Streptococcus* meningitis survivors Group B *Streptococcus* meningitis survivors followed up for median of ≥6 months with moderate-severe NDI where NMR <5 / 1000

Region	Country	Median year of data collection	Author	Number of GBS meningitis survivors	Number of GBS meningitis survivors with moderate-severe NDI	Neonatal mortality rate	ES (95% CI)	% Weight
Asia								
Southeastern Asia	Singapore	2005.5	Wee	20	3	1	0.15 (0.03, 0.38)	33.16
Subtotal (I-squared = .%, p = .)							0.15 (-0.02, 0.32)	33.16
.								
Developed								
Northern Europe	Sweden	1983	Bennhagen	9	1	4	0.11 (0.00, 0.48)	17.34
Northern Europe	UK	2015.5	Heath	37	8	2	0.22 (0.10, 0.38)	49.50
Subtotal (I-squared = 0.0%, p = 0.460)							0.19 (0.07, 0.31)	66.84
.								
Overall (I-squared = 0.0%, p = 0.713)							0.18 (0.08, 0.28)	100.00

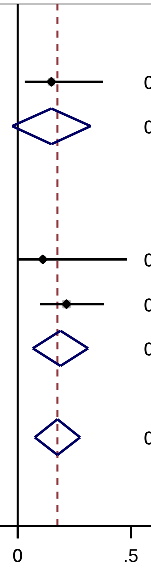

NOTE: Weights are from random effects analysis

ES=Effect size, NDI=Neurodevelopmental impairment

References

1. WHO. Clinical signs that predict severe illness in children under age 2 months: a multicentre study. Lancet 2008; 371(9607): 135-42.
2. Ku LC, Boggess KA, Cohen-Wolkowiez M. Bacterial Meningitis in the Infant. Clinics in perinatology 2015; 42(1): 29-45.
3. Mathur N, Garg K, Kumar S. Respiratory distress in neonates with special reference to pneumonia. Indian pediatrics 2002; 39(6): 529-38.
4. WHO. International Statistical Classification of Diseases and Related Health Problems 10th Revision. 2016.
5. Global Burden of Disease Study C. Global, regional, and national incidence, prevalence, and years lived with disability for 301 acute and chronic diseases and injuries in 188 countries, 1990-2013: a systematic analysis for the Global Burden of Disease Study 2013. Lancet 2015; 386(9995): 743-800.
6. Lawn JE, Blencowe H, Darmstadt GL, Bhutta ZA. Beyond newborn survival: the world you are born into determines your risk of disability-free survival. Pediatric Research 2013; 74(Suppl 1): 1-3.
7. WHO. International Classification of Functioning, Disability and Health. 2001.
8. Beery KE. Developmental test of visual-motor integration: Administration, scoring and teaching manual: Modern Curriculum Press, 1989.
9. Thompson G, Weber BA. Responses of infants and young children to behavior observation audiometry (BOA). Journal of Speech and Hearing Disorders 1974; 39(2): 140-7.
10. Albers CA, Grieve AJ. Test review: Bayley, N.(2006). Bayley scales of infant and toddler development–third edition. San Antonio, TX: Harcourt assessment. Journal of Psychoeducational Assessment 2007; 25(2): 180-90.
11. Achenbach TM, Edelbrock C. Child behavior checklist. Burlington (Vt) 1991; 7.
12. Ireton H. Child development inventory. Behavior Science Systems Minneapolis, MN, 1992.
13. Carrow E. A test using elicited imitations in assessing grammatical structure in children. Journal of Speech and Hearing Disorders 1974; 39(4): 437-44.
14. Cattell P. Cattell infant intelligence scale: Psychological Corporation, 1960.
15. Frankenburg WK, Dodds J, Archer P, Shapiro H, Bresnick B. The Denver II: a major revision and restandardization of the Denver Developmental Screening Test. Pediatrics 1992; 89(1): 91-7.
16. Michalec D, Henninger N. Expressive One-Word Picture Vocabulary Test. Encyclopedia of Clinical Neuropsychology: Springer, 2011:1000-2.
17. Merker B, Podell K. Grooved pegboard test. Encyclopedia of clinical neuropsychology: Springer, 2011:1176-8.
18. Kirk SA, McCarthy JJ. The Illinois Test of Psycholinguistic Abilities: An approach to differential diagnosis. American Journal of Mental Deficiency 1961.
19. Ireton H, Thwing E. Minnesota child development inventory: Behavior Science Systems Minneapolis, 1974.
20. Hellbrügge T. Münchener funktionelle Entwicklungsdiagnostik, 1994.
21. McCarthy D. McCarthy scales of children's abilities: Psychological Corporation, 1972.
22. Mullen EM. Mullen scales of early learning: AGS Circle Pines, MN, 1995.
23. Glascoe FP. Parents' evaluation of developmental status (PEDS). Ellsworth & Vandermeer Press, Ltd 1997.
24. Zimmerman IL, Steiner VG, Pond RE. PLS-3: Preschool language scale-3: Psychological Corporation, 1992.
25. Dunn LM, Dunn LM, Bulheller S, Häcker H. Peabody picture vocabulary test: American Guidance Service Circle Pines, MN, 1965.
26. Heller MF, Anderman BM, Singer EE. Pure Tone Audiometry. Functional Otology: Springer, 1955:45-64.
27. Thorndike RL, Hagen EP, Sattler JM. Stanford-Binet intelligence scale: Riverside Publishing Company, 1986.

28. Walker G, Dillon H, Byme D. Sound field audiometry: recommended stimuli and procedures. Ear and Hearing 1984; 5(1): 13-21.
29. Ferris FL, Kassoff A, Bresnick GH, Bailey I. New visual acuity charts for clinical research. American journal of ophthalmology 1982; 94(1): 91-6.
30. Hresko WP, Reid DK, Hammill DD. Test of early language development (TELD-3). Austin, TX: Pro-Ed 1999.
31. Sparrow SS, Balla DA, Cicchetti DV, Harrison PL, Doll EA. Vineland adaptive behavior scales. 1984.
32. Wechsler D. Wechsler individual achievement test. San Antonio, TX: Psychological Corporation, 1992.
33. Wechsler D. Manual for the Wechsler intelligence scale for children, revised: Psychological Corporation, 1974.
34. Woodcock RW, Johnson MB, Mather N. Woodcock-Johnson psycho-educational battery--Revised: DLM Teaching Resources, 1990.
35. Wechsler D. Manual for the Wechsler preschool and primary scale of intelligence: Psychological Corporation, 1967.
36. Jastak S, Wilkinson GS. The wide range achievement test-revised: Jastak Associates, 1984.
37. Horn KA, Zimmerman RA, Knostman JD, Meyer WT. Neurological sequelae of group B streptococcal neonatal infection. Pediatrics 1974; 53(4): 501-4.
